# Brain activation and connection across resting and motor‐task states in patients with generalized tonic–clonic seizures

**DOI:** 10.1111/cns.14672

**Published:** 2024-04-21

**Authors:** Sisi Jiang, Yuehan Wang, Haonan Pei, Hechun Li, Junxia Chen, Yutong Yao, Qifu Li, Dezhong Yao, Cheng Luo

**Affiliations:** ^1^ The Clinical Hospital of Chengdu Brain Science Institute, MOE Key Lab for Neuroinformation, School of Life Science and Technology, University of Electronic Science and Technology of China Chengdu P. R. China; ^2^ Research Unit of NeuroInformation Chinese Academy of Medical Sciences Chengdu P. R. China; ^3^ High‐Field Magnetic Resonance Brain Imaging Key Laboratory of Sichuan Province Center for Information in Medicine University of Electronic Science and Technology of China Chengdu P. R. China; ^4^ Department of Neurosurgey Sichuan Provincial People's Hospital, University of Electronic Science and Technology of China Chengdu P. R. China; ^5^ Department of Neurology Hainan Medical University Hainan P. R. China

**Keywords:** activation, fMRI, generalized tonic–clonic seizures, motor‐task state, network connectivity, resting state, white matter

## Abstract

**Aims:**

Motor abnormalities have been identified as one common symptom in patients with generalized tonic–clonic seizures (GTCS) inspiring us to explore the disease in a motor execution condition, which might provide novel insight into the pathomechanism.

**Methods:**

Resting‐state and motor‐task fMRI data were collected from 50 patients with GTCS, including 18 patients newly diagnosed without antiepileptic drugs (ND_GTCS) and 32 patients receiving antiepileptic drugs (AEDs_GTCS). Motor activation and its association with head motion and cerebral gradients were assessed. Whole‐brain network connectivity across resting and motor states was further calculated and compared between groups.

**Results:**

All patients showed over‐activation in the postcentral gyrus and the ND_GTCS showed decreased activation in putamen. Specifically, activation maps of ND_GTCS showed an abnormal correlation with head motion and cerebral gradient. Moreover, we detected altered functional network connectivity in patients within states and across resting and motor states by using repeated‐measures analysis of variance. Patients did not show abnormal connectivity in the resting state, while distributed abnormal connectivity in the motor‐task state. Decreased across‐state network connectivity was also found in all patients.

**Conclusion:**

Convergent findings suggested the over‐response of activation and connection of the brain to motor execution in GTCS, providing new clues to uncover motor susceptibility underlying the disease.

## Graphical abstract

Motor susceptibility in patients with generalized tonic‐clonic seizures.

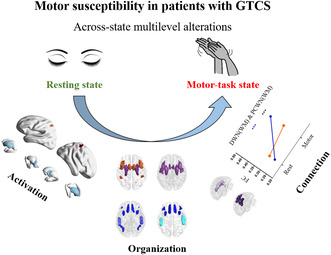

## 1 INTRODUCTION

Idiopathic generalized epilepsy (IGE) is a group of epileptic disorders characterized by interictal generalized spike waves in electroencephalography (EEG) without any evidence of brain lesions.^1^ Generalized tonic–clonic seizures (GTCS) are a common subtype of IGE resulting in serious clinical manifestations.^2^ The heterogeneous syndromes differ greatly in terms of clinical manifestation and prognosis in patients with IGE.^3^ Although patients do not show obvious motor symptoms during the interictal period, a large number of studies have found hyper‐excitability and hyper‐sensitivity of the motor system in IGE,^4^, ^5^ and inferred that the susceptibility of the motor system might be an important factor for the instability of the epileptic brain state.

In one recent resting‐state MRI study, Zhang et al., reported increased functional and structural connectivity within the sensorimotor network (SMN) in patients with GTCS, supporting the susceptibility of the motor system.^6^ Cognitive and motor conditions have been found to contribute to inducing epileptic discharges in some patients with epilepsy.^7^ Besides, previous studies have demonstrated that patients with IGE showed increased co‐activation of primary and supplementary motor areas (SMA) when performing cognitive tasks, which was inferred to be a possible mechanism for the cognition‐induced motor symptom.^8^ Many previous studies investigated the pathomechanism of brain networks of epilepsy using resting‐state fMRI,^9^, ^10^, ^11^ but fewer studies concerned brain networks in the motion state. It is generally believed that the resting state reflects the basic brain state,^12^ while the motor state reflects a primary “activation” state.^13^ The motor system plays an important role in epilepsy, thus focusing on the characteristics of the interaction between brain networks in a motion state might provide a cue to manifest in the resting state. Furthermore, identifying activation patterns and network changes during state transition might uncover novel evidence to understand the pathomechanism.

Functional connectivity (FC) captures the statistical dependence or temporal correlations between different regions.^14^, ^15^ The blood oxygen level‐dependent signal was previously known to reflect neural activity primarily in the gray matter, thus previous studies investigated FC between gray‐matter networks. In recent years, more and more attention has been paid to the functional characteristics of white matter. Ding and colleagues detected synchronous brain activity in white matter tracts at rest and under functional tasks.^16^ Subsequently, the functional activity of white matter has been confirmed by many task‐state studies, including perceptual, language, and motor tasks.^17^, ^18^, ^19^ Besides, Peer et al. divided all white matter into several functional clusters and found concordance between white‐matter FC and structural connectivity defined by diffusion tensor imaging, which further suggested inherent functional organization within the white matter.^20^ Moreover, the white‐matter FC has been recognized to be related to the pathomechanism of several mental disorders, such as schizophrenia and Alzheimer's disease.^21^, ^22^ Specifically, the study of white matter function in epilepsy has been gradually carried out. Some studies have uncovered disrupted white‐matter FC in patients with epilepsy and demonstrated the great potential of researching white‐matter profiles.^23^, ^24^, ^25^ Taken together, incorporating white‐matter functional networks into the study of epileptic brain networks is helpful to reveal the pathological mechanism of the disease, especially in combination with the characteristics of different brain states.

In this study, we collected fMRI data of resting and motor‐task states in patients with GTCS and healthy controls. First, motor activation and its association with head motion and cerebral functional gradients were accessed. Then, we investigated the FC between white‐matter and gray‐matter networks in resting and motor‐task states in patients with GTCS. Moreover, to further identify network characteristics related to the state transition, within‐ and between‐network similarity of connective pattern and temporal correlation across states were accessed. The present study aims to recognize brain activation and network interaction profiles across resting and motor‐task states and hypothesize that the motor‐task state might help to uncover the mechanisms underlying motor susceptibility in GTCS.

## 2 METHODS

### 2.1 Participants

This study recruited 50 patients with GTCS and 52 matched healthy controls (HC). All patients were diagnosed according to the epilepsy classification of the International League Against Epilepsy.^1^, ^26^ In the present study, 18 patients with GTCS (13 males, age: 24.8 ± 9.5 years) were newly diagnosed without antiepileptic drugs (ND_AEDs) and 32 patients were receiving AEDs (15 males, age 24.6 ± 4.8 years) (AEDs_GTCS). No participants had brain lesions, developmental disabilities, or other accompanying neurological disorders. All participants are right‐handed. Detailed demographic and clinical information are shown in Table 1. This study was approved by the ethical committee of the University of Electronic Science and Technology of China according to the standards of the Declaration of Helsinki, and written informed consent was obtained from all participants.

**TABLE 1:** Demographics of subjects in the present study.

Characteristics	HC (mean ± SD)	GTCS (mean ± SD)	*t*/*χ* ^2^	*p*
Simple size	52	50	–	–
Age (years)	23.98 ± 5.222	24.74 ± 6.842	0.631	0.529
Gender (female/male)	21/31	22/28	0.137	0.712
Duration (years)	–	5.20 ± 5.326	–	–
Therapy (naïve: drug)	–	18/32	–	–
mFD_rest	0.07 ± 0.02	0.12 ± 0.06	4.61	<0.001
mFD_motor	0.10 ± 0.05	0.27 ± 0.18	6.33	<0.001

Abbreviations: mFD, mean frame‐wise displacement; mFD_rest, mFD in the resting state; mFD_motor, mFD in the motor state.

### 2.2 Data acquisition

#### 2.2.1 Resting‐state fMRI

All subjects underwent MRI scanning in a 3 T GE scanner with an eight‐channel‐phased array head coil (EXCITE, GE, Milwaukee, WI) in the information medical center of the University of Electronic Science and Technology. Functional data were collected using an echo‐planar imaging sequence with the following parameters: repetition time (TR) = 2000 ms, echo time (TE) = 30 ms, flip angle (FA) =90°, slice thickness = 4 mm (no gap), data matrix = 64 × 64, field of view = 24 cm × 24 cm, voxel size = 3.75 × 3.75 × 4 mm^3^, and 32 axial slices in each volume. Two hundred and fifty‐five volumes were acquired in each scan. Axial anatomical T1‐weighted images were acquired using a 3‐dimensional fast spoiled gradient echo sequence. The parameters were as follows: thickness = 1 mm (no gap), TR = 8.2 ms, TE = 3.2 ms, field of view = 25.6 cm × 25.6 cm, flip angle = 12°, data matrix = 256 × 256. There were 136 axial slices for each subject. During the resting state, all subjects were instructed to close their eyes and relax without falling asleep during the scan.

#### 2.2.2 Motor‐task state fMRI

We designed a hand‐moving motor task with a length of 610 s. Participants lie in the scanner with a screen in front of his/her eyes. All participants were instructed to move their left hand when they saw an arrow on a screen pointing to the left and their right hand when they saw an arrow pointing to the right. The participants were asked to flip their wrists back and forth at a rate of 1 s, similar to bouncing a ball. This task consisted of 20 blocks, each lasting 16 s with a 14‐s interval between blocks. The experiment was preceded by a 10‐s message. Three hundred and five volumes were acquired in each scan. The fMRI data were acquired using the parameters mentioned above.

### 2.3 Data preprocessing

Structural T1 images were segmented into gray matter, white matter, and cerebrospinal fluid. Using SPM8 and NIT toolbox,^27^ the first five volumes of fMRI data were discarded, and slice‐timing, realignment, and co‐registration with anatomical images were performed subsequently. Then, the linear trend, 24 head motion parameters, and cerebrospinal fluid signal were regressed from fMRI data. Besides, a band filter (0.01–0.10 Hz) was performed to reduce the non‐neuronal signals. The mean frame‐wise displacement (mFD) and the mean translation and rotation were calculated and compared between patients and healthy controls. Notably, we employed the Jarque–Bera test to assess the normality of the data (*p* < 0.05). Parametric tests were conducted for data conforming to a normal distribution, while non‐parametric tests were applied to data not conforming to a normal distribution. For data conforming to a normal distribution, we employed two‐sample t‐tests, parametric one‐way analysis of variance (ANOVA) to detect between‐group differences with age, gender, and mFD as covariates. For data not conforming to a normal distribution, we utilized the Mann–Whitney U test to conduct a non‐parametric independent two‐sample test and Kruskal–Wallis test for non‐parametric one‐way ANOVA. In this study, linear mixed model based on the statistic codes in DPABI toolbox (https://rfmri.org/DPABI) was employed for two‐way repeated ANOVA with age, gender, and mFD as covariates. All subsequent statistical methods adhere to this principle.

Notably, to avoid signal mixing, the functional images were smoothed separately in gray matter and white matter (4 mm full‐width half‐maximum). For each subject, the identification of gray‐ and white‐matter voxels depended on the segmented images using a threshold of 0.5. Thus, we obtained one image only containing white‐matter voxels and one image only containing gray‐matter voxels. By combining smoothed white‐matter images and gray‐matter images, full functional images were finally obtained for each subject. These functional images were normalized to the Montreal Neurological Institute EPI template and resampled to 3 mm cubic voxels. Group‐level white‐matter and gray‐matter masks were constructed by retaining voxels that are present in 60% and 20% of the individuals, respectively.^28^ Subcortical regions from the Harvard–Oxford Atlas (including the thalamus, putamen, caudate, and accumbens) were removed from the group‐level white‐matter mask. Finally, we obtained a white‐matter mask consisting of 18,591 voxels.

### 2.4 Motion activation

Using SPM12 (https://www.fil.ion.ucl.ac.uk/spm/software/spm12/), a general linear model consisting of a boxcar regressor with two levels (motor execution and fixation) and convolved with a classical hemodynamic response function. Within‐ and between‐group differences of activation maps were detected in this work. Furthermore, we calculated the correlation between motion activation and head motion during motion execution using Pearson's correlation. Based on the previously proposed network atlas,^20^ we calculated the averaged correlation coefficients in nine recognized gray‐matter functional networks, which are identified using a clustering approach. The correlation coefficients were Fisher‐z transformed. Nine gray‐matter functional networks consisted of a sensorimotor network (SMN), frontoparietal control network (FPCN), default mode network (DMN), ventral attention network (VAN), dorsal attention network (DAN), cerebellum posterior network (CPN), cerebellum anterior network (CAN), temporal‐orbitofrontal network (TON), and visual network (VN). The correlation between motor task activation and head motion was compared between patients and healthy controls.

Moreover, to access the association between activation and intrinsic brain functional hierarchy, we also conducted spatial correlation analysis with cerebral functional gradients.^29^ To account for spatial auto‐correlation, a spin test based on spherical rotations (5000 times) was used for the inferential analysis of the correlation results (permutation test).^30^ The spin test generates surrogate brain maps that are matched to an empirical brain for spatial autocorrelation, thus controlling for spatial contiguity and hemispheric symmetry. To compare the association between groups, we further performed the non‐parametric permutation statistics by randomly shuffling subjects 5000 times and constructed a null model of spatial correlation between activation maps and cerebral functional gradients.

### 2.5 Functional connectivity between white‐ and gray‐matter networks

#### 2.5.1 White‐matter and gray‐matter networks

In this study, we recognized white‐matter networks using the fMRI data in both states according to our previous study.^28^ To reduce computational complexity, the 18,591 white‐matter voxels were subsampled to 4623 nodes based on an interchanging grid strategy.^31^ Then, we calculated the Pearson's correlation coefficient between all white‐matter voxels and subsampled nodes, resulting in 18,591 × 4623 correlation matrices for each subject. A K‐means clustering approach was performed on an averaged correlation matrix across all subjects, which was replicated 10 times. The number of clusters ranges from 2 to 22. To verify the stability of clustering results, we randomly divided the averaged 18,591 × 4623 correlation matrix into four folds with the size of 18,591 × 1155 and performed the clustering analysis in each fold. Adjacency matrices were computed for clustering results in every fold, and Dice's coefficient was computed for two adjacency matrices to access the similarity of clustering results. Averaged Dice's coefficient was used to reflect the stability of clustering results. Specifically, to investigate the consistency of white‐matter networks across states, we also clustered white‐matter networks in the resting and motor‐task states separately.

Moreover, we clustered white‐matter functional networks using resting‐state fMRI data, and motor‐task state fMRI data, respectively, resulting in white‐matter functional networks in each state. To access the potential reorganization of networks, we calculated the dice coefficients of spatial patterns between networks. We assumed that the spatial reorganization of networks might be caused by changes in time dependence. Thus, we explored the higher‐order connectivity between networks in different states. Similarly, rANOVA was performed to investigate between‐group differences with age, gender, and mFD as the covariates.

Many previous studies have divided the gray matter into several functional networks with great reproducibility.^31^, ^32^ In this study, we enrolled nine recognized gray‐matter functional networks, which are identified using a clustering approach similar to the approach used in the present study.^20^ Nine gray‐matter functional networks consisted of a sensorimotor network (SMN), frontoparietal control network (FPCN), default mode network (DMN), ventral attention network (VAN), dorsal attention network (DAN), cerebellum posterior network (CPN), cerebellum anterior network (CAN), temporal‐orbitofrontal network (TON), and visual network (VN).

#### 2.5.2 Within‐state connectivity analysis

In each subject, for each white‐matter and gray‐matter network, we extracted the averaged time courses of all within‐network voxels. Pearson's correlation coefficients were calculated between the time courses of pairs of networks. A Fisher's Z transformation was used to normalize the correlation coefficients. Thus, for each subject, two network correlation matrices were generated for the resting and motor‐task states, respectively. To identify significant between‐network connectivity within the group, one‐sample t‐tests were performed in both groups in resting and motor‐task states. Then, for the FC between white‐matter and gray‐matter networks, two‐way rANOVA test were used to detect the interaction effects of group and state. Besides, post hoc tests were conducted to detect specific between‐group differences.

#### 2.5.3 Across‐state connectivity analysis

In addition to the FC within each state, we also investigated the across‐state functional interaction using the across‐state functional connectivity (AS‐FC) between white‐matter networks. We deleted the last 45 data points of the motor‐task state data to maintain the same length (250 points) as the resting‐state data. The AS‐FC was calculated by Pearson's correlation coefficient of the time course of networks between states, which is described as follows:

$$\mathrm{AS}-{\mathrm{FC}}_{i,j}=\text{Corr}\;\left({\mathrm{TC}}_{\text{motor}}\left(i\right)-{\mathrm{TC}}_{\text{rest}}\left(j\right)\;\right),$$

where the ${\mathrm{TC}}_{\text{motor}}\left(i\right)$ represents the time course of network i in the motor‐task state and the ${\mathrm{TC}}_{\text{rest}}\left(i\right)$ represents the time course of network i in the resting state. To exclude the influence of the inherent correlation of the BOLD signal itself at different times on the cross‐state correlation, we further corrected the AS‐FC. We first calculated the Pearson's correlation coefficients between the first 125 time points and the last 125 time points of the time course of each network in the resting and motor‐task states, respectively, and took the absolute value to represent the inherent correlation of the BOLD signal itself. The AS‐FC value was then divided by the intrinsic correlation to obtain the corrected AS‐FC. Finally, the corrected AS‐FC values were z‐scored for statistical analysis. The white‐matter networks were identified in the resting state and motor‐task state, respectively.

Moreover, we also investigated across‐state higher‐order functional connectivity (AS‐HFC) between all networks was also investigated in this study. Because there are only 21 brain networks, the length of data used for AS‐HFC is relatively short (20 data points), and the Euclidean distance is more suitable for describing similarity than the Pearson correlation coefficient. Thus, we portrayed the AS‐HFC using the following formula:

$$\begin{array}{c} \begin{array}{c} \mathrm{AS}-{\mathrm{HFC}}_{i,j}=\sqrt{\sum_{\begin{array}{c} \;i=1\\ \;j=1 \end{array}}^{m}{\left({\mathrm{FCP}}_{\text{motor}}\left(i\right)-{\mathrm{FCP}}_{\text{rest}}\left(j\right)\right)}^{2}}, \end{array} \end{array}$$

where the ${\mathrm{FCP}}_{\text{motor}}\left(i\right)$ represents the functional connectivity pattern of network i in the motor‐task state, the functional connectivity pattern is a vector consisting of FC values between network i and all other networks. Similarly, the ${\mathrm{FCP}}_{\text{rest}}\left(i\right)$ represents the functional connectivity pattern of network i in the resting state. Lower AS‐HFC indicates higher across‐state similarity. To avoid a misunderstanding of intuitive concepts, we scaled the AS‐HFC to the range of 0–1 and then subtracted from 1, thus higher scaled AS‐HFC indicates higher similarity. Parametric (or non‐parametric) two‐sample and ANOVA tests were used to investigate the significant differences of $\mathrm{AS}-{\mathrm{FC}}_{i,j}$ and $\mathrm{AS}-{\mathrm{HFC}}_{i,j}$ between patients and control, and between subgroups.

## 3 RESULTS

### 3.1 Head motion and activation

Using 2 × 2 rANOVA, we found a significant interaction of head motion between groups and states (FDR *p* < 0.05) (Figure S1). Both in the resting and motor states, patients with GTCS showed significantly higher head motion than the HC (*p* < 0.001). Moreover, the patients showed increased head motion in the motor state than in the resting state (Figure S1A). The patients did not show different head rotations in the resting state. In the subgroup analysis, we also found that both the ND_GTCS and AEDs_GTCS showed greater head motion than controls and increased mean FD in motor states than the resting state (Figure S1B). Notably, the AEDs_GTCS showed a trend toward greater head movement in ND_GTCS, although it did not reach a statistically significant level. The ND_GTCS even did not show the difference in head translation and rotation relative to the HC.

**FIGURE 1:**
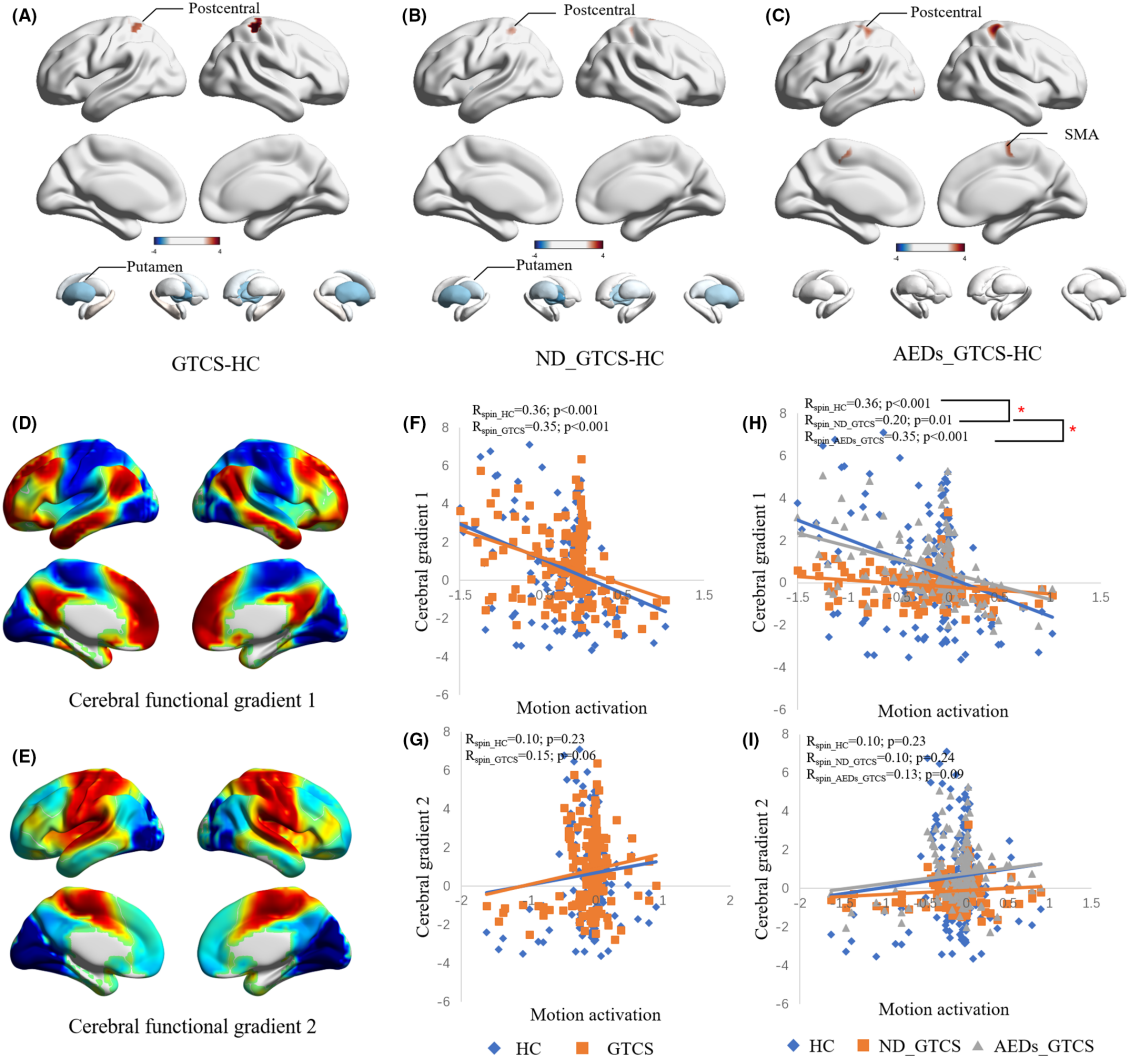
Comparisons of motor task activation and its spatial correlation with functional gradients. (A–C) Group comparisons between patients with GTCS and healthy controls, and between subgroups of patients. (D, E) Functional gradient maps from our previously published work. (F, G) Spatial correlation in HC and GTCS. (H, I). Spatial correlation in HC, AEDs_GTCS, and ND_GTCS. The read star (*) indicates *p* < 0.05.

During the motion execution, all patients showed similar activation patterns, activating the sensorimotor cortex and deactivation the default mode network (DMN) (Figure S2). Compared with the HC, patients with GTCS showed increased activation in the postcentral and decreased activation in the bilateral putamen (*p* < 0.001, Figure 1A). The ND_GTCS also had similar alterations of activation as the whole patient group (*p* < 0.001, Figure 1B). Interestingly, AEDs_GTCS showed extra increased activation in the SMA and did not show abnormal activation in the subcortical regions (Figure 1C, Table S1). No differences were observed between AEDs_GTCS and ND_GTCS.

The cerebral functional gradients are from our previously published work (Figure 1D, E). Both the HC and GTCS showed a significantly negative correlation with cerebral gradient 1 (*p*_spin_ <0.001) (Figure 1F). The ND_GTCS showed a decreased association between motion activation and cerebral gradient 1 than the HC and AEDs_GTCS (*p*_perm_ <0.001) (Figure 1G). No correlation was found between motion activation and cerebral gradient 2 in all groups (Figure 1H, I).

The patients with GTCS did not show a different association between head motion in the motor state and motion activation (Figure S3A). In the subgroup analysis, compared with the HC and AEDs_GTCS (Figure S3B), the ND_GTCS showed a great association between head motion and activation in the VN, CPN, VAN, DMN, and FPCN. The AEDs_GTCS did not show a difference with HC.

### 3.2 White‐matter networks

**FIGURE 2:**
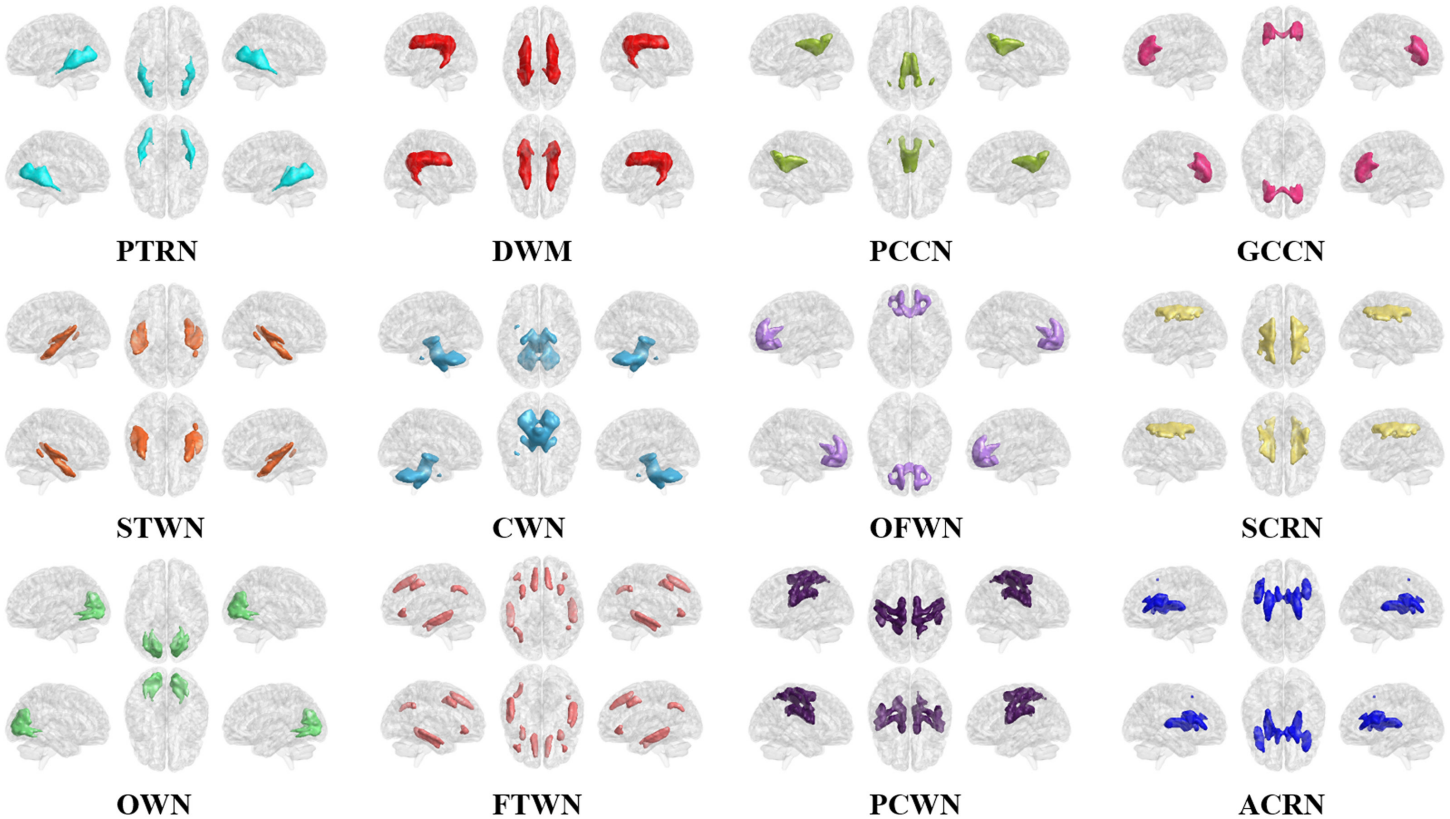
Twelve white‐matter networks clustered using resting and motor‐task state fMRI together. ACRN, anterior corona radiate network; CWN, cerebellar white‐matter network; DWN, deep white‐matter network; FTWN, frontotemporal white‐matter network; GCCN, genu of corpus callosum network; OFWN, orbitofrontal white‐matter network; OWN, occipital white‐matter network; PCCN, posterior corpus callosum network; PCWN, precentral/postcentral white‐matter network; PTRN, posterior thalamic radiation network; SCRN, superior corona radiate network; STWN, Superior temporal white‐matter network.

In this study, the K‐means algorithm was used to cluster white‐matter networks, and the similarity of clustering results was measured by the Dice coefficient. The results showed that *K* = 12 was the maximum K value with a Dice coefficient of >0.89, and 12 white‐matter networks were finally obtained (Figure 2). We named these networks according to their spatial location and divided them into three layers (deep, middle, and superficial) (Table S2).

Additionally, we also clustered white‐matter networks using resting‐state and motor‐task state fMRI data, respectively, and obtained 13 white‐matter networks (Figure S4), including 11 extremely similar networks. Notably, we found that the ACRN existed as one network in the motor‐task state, and divided into two subnetworks in the resting state. Besides, an internal capsule/cerebral peduncle network (ICPN) was identified in the motor‐task state. To distinguish the white matter networks in the resting state from the motor‐task state, we added the letters “r” and “m” before the names of the brain networks, respectively.

### 3.3 Alterations of FC between networks in GTCS in different states

**FIGURE 3:**
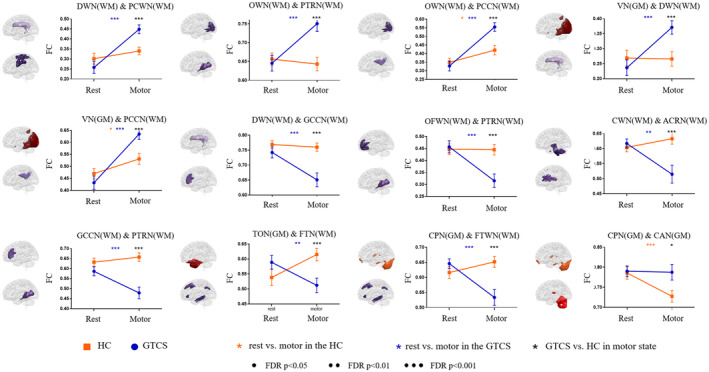
Connections with interaction effects of group and state. The yellow star represents the comparison between the resting and motor‐task states in the HC, the blue star represents the comparison between the resting and motor‐task states in the GTCS, and the black star represents the comparison between GTCS and HC in the motor‐task state. In subgroup analysis, both the ND_GTCS and AEDs_GTCS showed significant alterations in network connectivity between states with the same trend, including five increased connections and three decreased connections.

We found that 12 connections showed a significant interaction between the group and states (Figure 3 and Figure S5) (*p* < 0.05, FDR corrected). Compared with HC, patients with GTCS showed a greater increase in FC in five connections from the resting state to the motor‐task state, including three white‐matter connections between DWN and PCN, between OWN and PTRN, between OWN and PCCN, and one connection between gray‐matter VN and white‐matter DWN. Besides, patients with GTCS also showed a greater decrease in six connections relative to HC, including white‐matter connections between CWN and ACRN, between DWN and GCCN, between CPN and FTWN, between OFWN and PTRN, and between GCCN and PTRN, and one connection between gray‐matter TON and white‐matter FTWN. Moreover, the connection between gray‐matter CPN and CAN showed decreased connectivity in the motor‐task state relative to the resting state, in which patients did not show significant change. Post hoc analyses showed that among the 12 connections with interaction effects, the motor task induced significant changes in 11 connections in patients relative to the resting state, while only three connections were changed in the healthy controls. In particular, we found no significant between‐group differences in the resting state, but significant differences in all connections were found in the motor‐task state.

In subgroup analysis, there are eight connections that showed significant interactions of group and state (Figure S6 and S7) (*p* < 0.05, FDR corrected). In all connections, the ND_GTCS and AEDs_GTCS showed similar changes between states. The connection between PTRN and VN and the connection between PTRN and STWN were decreased in ND_GTCS in the resting state and increased in AEDs_GTCS in the motor state.

### 3.4 White‐matter functional network re‐organization

**FIGURE 4:**
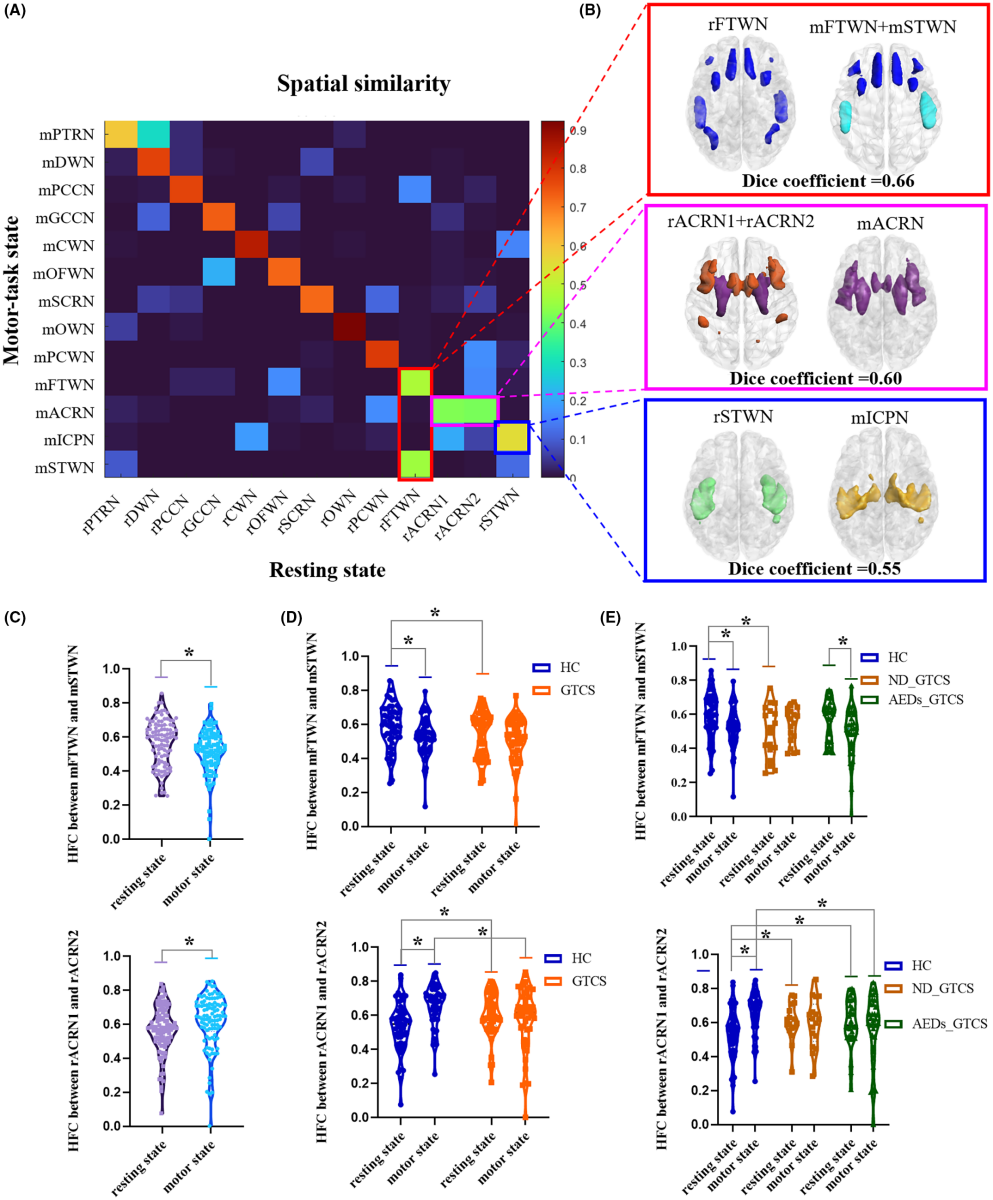
The similarity between white‐matter networks clustered resting and motor‐task states. (A) The spatial similarity between resting and motor‐task states. (B) The dice coefficient between white‐matter networks varies across states. (C) In all subjects, HFC between mFTN and mSTN, and HFC between rACRN1 and rACRN2 were compared between resting and motor‐task states. (D) FC comparisons between patients and controls between states. (E) HFC comparisons between subgroups of patients and controls between states. The stars (*) represent significant between‐group differences.

Most white‐matter networks showed high spatial similarity across states, except for the mFTWN, mSTWN, mICCN, mACRN, rFTWN, rACRN1, rACRN2, and rSTWN (Figure 4A). To further clarify the network variance across states, we combined networks not consistent across states and tested the spatial similarity. We found that the FTWN acts as one whole in the resting state and was divided into two subnetworks in the prefrontal frontal and temporal in the motor‐task state. The rACRN1 and rACRN2 in the resting state merged into one network in the motor state. The rSTWN showed a dice coefficient of 0.55 with mICPN (Figure 4B). We also found a higher HFC between mFTWN and mSTWN in the resting state relative to the motor‐task state (*p* < 0.05). Higher HFC between rACRN1 and rACRN2 was found in the motor‐task state (*p* < 0.01) (Figure 4C). Patients showed lower HFC between mFTWN and mSTWN in the resting state relative to the HC. Meanwhile, the HFC between ACRN1 and ACRN2 in patients was higher in the resting state and lower in the motor‐task state than in healthy controls (Figure 4D) (*p* < 0.01). There was a significant change in HFC between mFTWN and mSTWN in AEDs_GTCS, but not in ND_GTCS (*p* < 0.01). The ND_GTCS showed deceased HFC between mFTWN and mSTWN in the resting state relative to the HC. The alterations of HFC between rACRN1 and rACRN2 relative to the HC were similar in both patient groups, except that ND_GTCS showed decreased HFC in the motor state (*p* < 0.05) (Figure 4E).

### 3.5 Alterations of across‐state connectivity in GTCS

**FIGURE 5:**
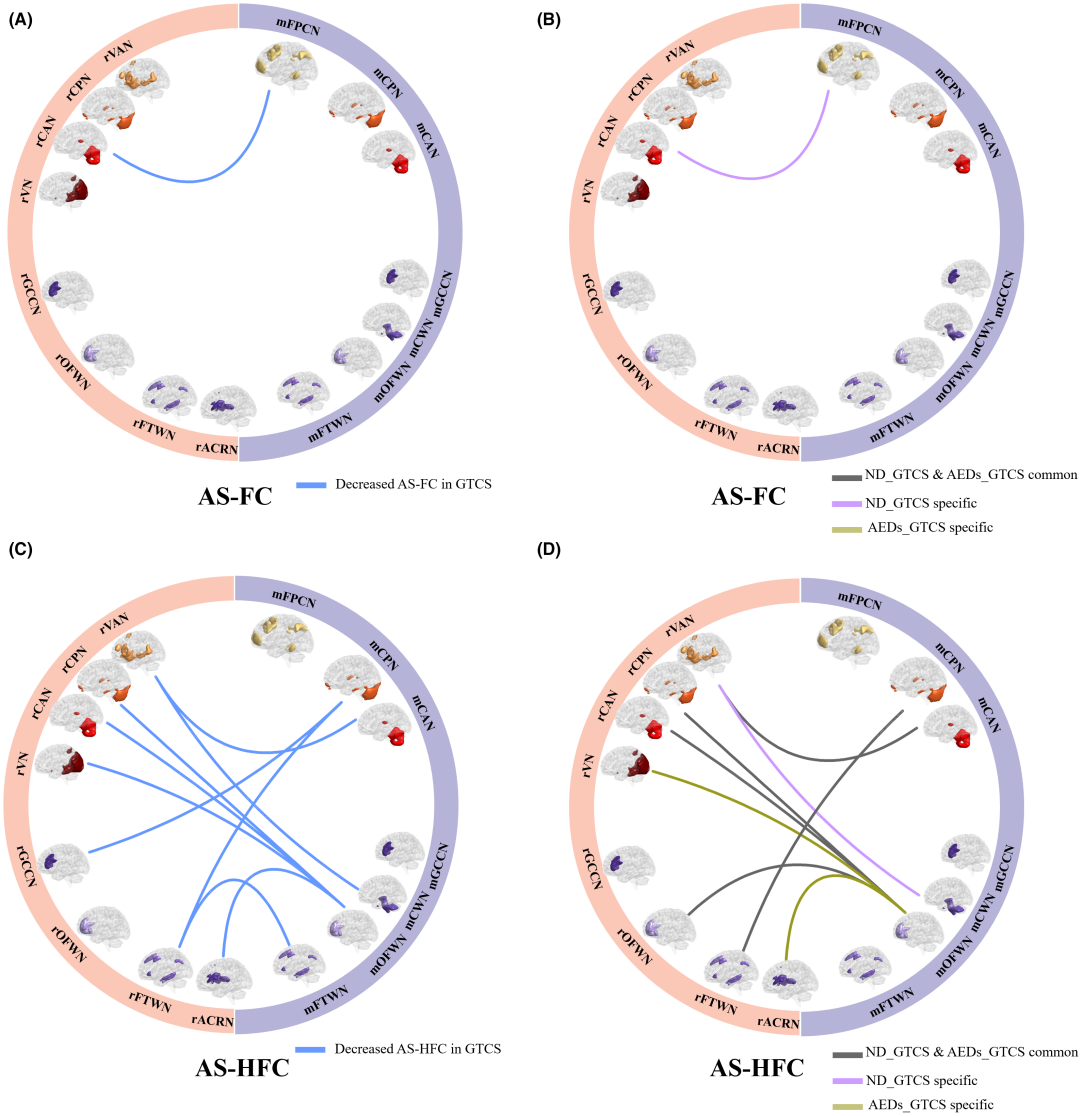
Across‐state connectivity in patients relative to the HC (*p* < 0.001). (A) Comparisons of AS‐FC between GTCS and HC. (B) Comparisons of AS‐FC between ND_GTCS, AEDs_GTCS, and HC. (C) Comparisons of AS‐HFC between GTCS and HC. (D) Comparisons of AS‐HFC between ND_GTCS, AEDs_GTCS, and HC.

Compared with HC, patients with GTCS showed decreased AS‐FC in one connection between CAN and FPCN (Figure 5A, Table S3) (*p* < 0.001, uncorrected). In further subgroup analysis, only ND_GTCS showed decreased AS‐FC between rCAN and mPFCN (Figure 5B, Table S3). Besides, decreased AS‐HFC, which major located links between gray matter and white matter networks, was observed in patients with GTCS (*p* < 0.001, uncorrected), including two connections linking the mCPN with rGCCN and rFTWN, one connection liking the mCAN and rVAN, one connection linking the rCWN with rVAN, four connections linking the mOFWN with rCAN, rVN, rOFWN, and rACRN, and one connection linking the mFTWN and rFTWN (Figure 5C, Table S4). In the subgroup analysis, both the ND_GTCS and AEDs‐GTCS showed decreased AS‐HFC relative to the HC, including five common connections in both groups, one specific connection in ND_GTCS, and two specific connections in AEDs‐GTCS (Figure 5D, Table S5).

## 4 DISCUSSION

In this study, we investigated brain activation and network connection in patients with GTCS. First, increased cortical activation in the postcentral gyrus was found in all patients and decreased subcortical putamen were observed in ND_GTCS. The activation of ND_GTCS also showed predominantly associated with head motion and decreased association with cerebral gradient 1. Moreover, all patients showed altered FC in motor‐task states, involving distributed gray‐matter cortical networks and deep‐middle white‐matter networks and cerebellum, such as corpus callosum and corona radiate. The AEDs_GTCS showed specifically higher increased FC between the posterior thalamic radiation network and visual and superior temporal white‐matter functional networks. All patients also showed decreased across‐state connectivity relative to the control, suggesting a significant variance in cerebellar, perceptual, and frontotemporal network interactions across states. Taken together, the present study provided evidence to uncover the motor susceptibility underlying the disease.

Motor abnormality is one of the typical clinical symptoms, which has been widely investigated in previous studies.^33^ We found higher head motion of patients than the healthy controls in both states and abnormally increased head motion in motor tasks than the resting state, suggesting over‐sensitivity of motion in patients. Notably, there was a tendency for greater head motion in AEDs_GTCS than in ND_GTCS. The higher head motion provided simple and direct behavior evidence to support motor sensitivity in patients with GTCS. Moreover, the hyper‐excitability of motor cortices has been verified by transcranial magnetic stimulation studies, and has been suggested to contribute to motor susceptibility.^4^, ^34^ Consistent with previous task‐fMRI studies, patients with epilepsy showed increased activation in the cortical sensorimotor cortices in the motor‐task state.^35^ Subcortical regions are also involved in the motor execution process.^36^ Impaired cortico‐striatal excitatory transmission is proposed to be a plausible mechanism that triggers epilepsy.^37^ In this study, decreased activation in the putamen was also found in the ND_GTCS, but not in the AEDs_GTCS, which might reflect the effects of AEDs on the epileptic brain.^38^ Compared with HC and AEDs_GTCS, we found the cortical activation abnormally negatively correlated with the head motion in ND_GTCS, further supporting disease‐related instability of the brain in the motor state. The cerebral functional gradient provides a characterization of the topographical organization of the cortical cortices and suggests a hierarchy from sensorimotor to the default mode network.^39^ The activation map of ND_GTCS showed a decreased association with cerebral gradient 1 compared to HC and AEDs_GTCS. This further indicates the disease might disrupt the functional hierarchical organization of the brain when processing motor information.

We also found that patients showed over‐increased FC between the deep white‐matter network and superficial primary motor white‐matter network in the motor‐task state relative to the resting state, which provided evidence indicating the involvement of white matter in the epileptic network.^23^, ^25^ Both the ND_GTCS and AEDs_GTCS showed similar changes in FC between resting and motor states. Vollmar et al., found that the primary and supplementary cortices would be activated when the patients with generalized epilepsy complete the working memory task, and the FC between the motor system and the frontoparietal cognitive network also abnormally increased.^8^, ^40^ In this study, we did not find increased connectivity between motor and cognitive gray‐matter networks, which might be because we used simple motor tasks that did not involve cognitive load. Our study found that the connectivity between the occipital white matter and multiple white‐matter networks increased in the motor‐task state relative to the resting state, which might be caused by the fact that the resting state in this study was acquired with eyes closed, and the motor‐task state induced visual stimuli. Specifically, the FC between the posterior thalamic radiation network and visual network, and superior temporal auditory networks was decreased in ND_GTCS in the resting state and increased in AEDs_GTCS in the motor state, suggesting the disrupted interaction between superficial primary information and deep networks, which might be responsible for the initial dysfunction of the disease.

A large‐sample multi‐center study suggested pronounced reductions in fractional anisotropy in the corpus callosum, and corona radiata in patients with generalized epilepsy.^41^ We found the motor task‐induced decreased FC between the corpus callosum and deep and posterior thalamic radiation networks, implying under‐integration within the deep‐middle white matter in patients. We hypothesized that patients with epilepsy might recruit other discretely distributed brain regions through the corpus callosum during the motor‐task state. A recently published study found a significant reduction of fractional anisotropy in the body of the corpus callosum in the group with lateralized seizures compared with the group with non‐lateralized seizures after corpus callosotomy, which also supported the hypothesis.^42^ Since generalized epilepsy itself is a neurological disease characterized by abnormal discharges propagating in wide brain cortices, the functional impairment of white matter tracts might be closely related to the pathological mechanism.^23^

It has been widely identified as a co‐activation of cerebral motor cortices and cerebellar cortices in multiple motor‐related tasks.^43^, ^44^ Specifically, the cerebellum has been recognized as a modulator of the epileptic network, affecting the thalamocortical and cortico‐cortical circuits.^45^, ^46^, ^47^ In this study, we found patients fail to decrease the FC within cerebellar networks while performing motor tasks, which might be related to the hyper‐connectivity in performing motor actions. The cerebellum has been found to connect with wide cerebral cortical networks and even has been divided into many subregions corresponding to common resting‐state brain networks.^48^ We also found the FC between posterior cerebellar gray matter and frontotemporal white matter of patients did not show differences with the controls in the resting state, whereas this connection was abnormally decreased in the motor‐task state. These findings further supported the contribution of the cerebellum to the motion susceptibility of patients.

The anterior corona radiate region appears as a whole network in the motor‐task state, while it is divided into two sub‐networks in the resting state. The anterior corona radiate region is in the pathways linking the thalamus and multiple cerebral cortices, playing important roles in the interaction between superficial and deep regions in motor, perceptual, and cognitive information processing.^49^, ^50^, ^51^ Our findings showed that purposeful motor execution could improve the overall functional consistency of the anterior corona radiate, which suggested the primary information integrating role of the anterior corona radiate. Moreover, the prefrontal and temporal white‐matter networks in the motor‐task state were divided into two networks in the motor state, which implied spatial reorganization in the task‐related network in the motor condition. Frontotemporal regions are involved in the majority of patients with generalized epilepsy,^52^, ^53^ which might lead to a fixed and over‐integrated network architecture in epilepsy. Here, we found that patients failed to flexibly change the network reorganization across resting and motor states, which might be related to the hyper‐integration of brain networks caused by disease.^54^ At the same time, we also discovered the instability of the patient's functional activity across states, which was found to be contrary to changes observed in spatial profiles of brain network. Distributed decreased across‐state connectivity in patients with GTCS also suggested the instability of the epileptic brain induced by motor tasks. Specifically, the cerebellum‐related connectivity predominantly contributed to the across‐state instability. Taken together, these findings provided evidence from network architecture and connectivity to suggest motor susceptibility in patients.

There are several limitations of the present study. First, our sample size is relatively small, and future studies need to use larger datasets to verify the stability of our findings. Although it is not yet clear what functional organization in white matter represents in a physiological sense, these findings from white matter may add an avenue to uncover brain mechanisms underlying the disease. The motor task in the present study is simple and more complex motor tasks should be designed to illustrate the motor susceptibility of patients.

## 5 CONCLUSION

This study first recognized abnormal motor activation and indicated increased activation in the cortical postcentral gyrus and decreased activation in the subcortical regions. Further exploring the association between activation and head motion and cerebral functional gradient suggested abnormally brain architecture during motor execution in patients with ND_GTCS. Moreover, whole‐brain network functional connectivity showed distributed abnormal connections in the motor‐task state in patients with GTCS, involving sensorimotor, cerebellum, and multiple other white‐matter networks, while no significant case–control differences in connectivity were observed in the resting state. Finally, decreased across‐state connectivity between networks was found in patients predominantly among gray‐matter cerebellum and distributed white‐matter networks. Taken together, these findings indicated that patients with GTCS showed abnormal motor task‐induced activation and network interactions, providing evidence to understand the motor susceptibility in patients.

## FUNDING INFORMATION

This work was supported by a grant from the STI 2030‐Major Projects 2022ZD0208500, the National Nature Science Foundation of China (U2033217, 61,933,003, 62,201,133, and 81,960,249), China Postdoctoral Science Foundation(2021TQ0061), Natural Science Foundation of Sichuan Province (2022NSFSC1320), and the CAMS Innovation Fund for Medical Sciences (CIFMS) (No.2019‐I2M‐5‐039).

## CONFLICT OF INTEREST STATEMENT

The authors report no competing interests.

## Supporting information


Data S1:

## Data Availability

The datasets used and/or analyzed during the current study are available from the corresponding author upon reasonable request.
