# Correction to *Brain activation and connection across resting and motor task states in patients with generalized tonic–clonic seizures*

**DOI:** 10.1111/cns.14790

**Published:** 2024-06-03

Jiang S, Wang Y, Pei H, et al. Brain activation and connection across resting and motor‐task states in patients with generalized tonic–clonic seizures. *CNS Neurosci Ther*. 2024;30:e14672. doi:10.1111/cns.14672

The affiliation information of author Qifu Li “Department of Neurology, Hainan Medical University, Hainan 571199, P. R. China” was incorrect.

The correct affiliation information for Qifu Li should be as follows: Department of Neurology, The First Affiliated Hospital of Hainan Medical University, Hainan 571199, P. R. China.

We apologize for this error.

