# Supplementary material for: Brain activation and connection across resting and motor‐task states in patients with generalized tonic–clonic seizures
Source: CNS Neurosci Ther. 2024 Apr 21;30(4):e14672. doi: 10.1111/cns.14672 (PMC11033329; doi:10.1111/cns.14672)
Supplement: Supplementary file 1 — Data S1: [file CNS-30-e14672-s001.docx]

**Supplementary materials for “*Brain activation and connection across resting and motor-task states in patients with generalized tonic-clonic seizures*”**


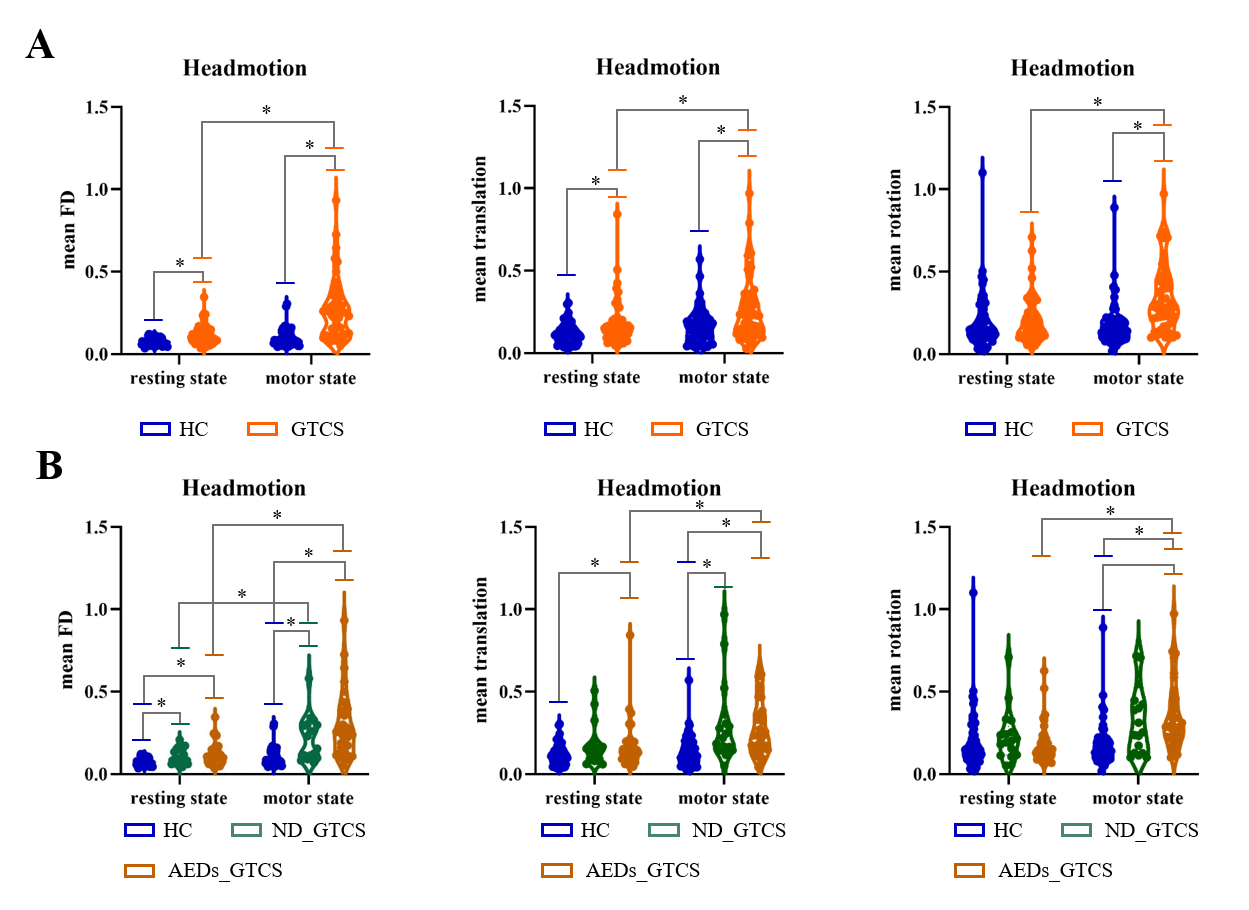


**Fig. S1** Head motion comparisons. (A) Head motion comparisons between patients with GTCS and healthy controls. (B) Head motion comparisons among ND_GTCS, AEDs_GTCS, and healthy controls.


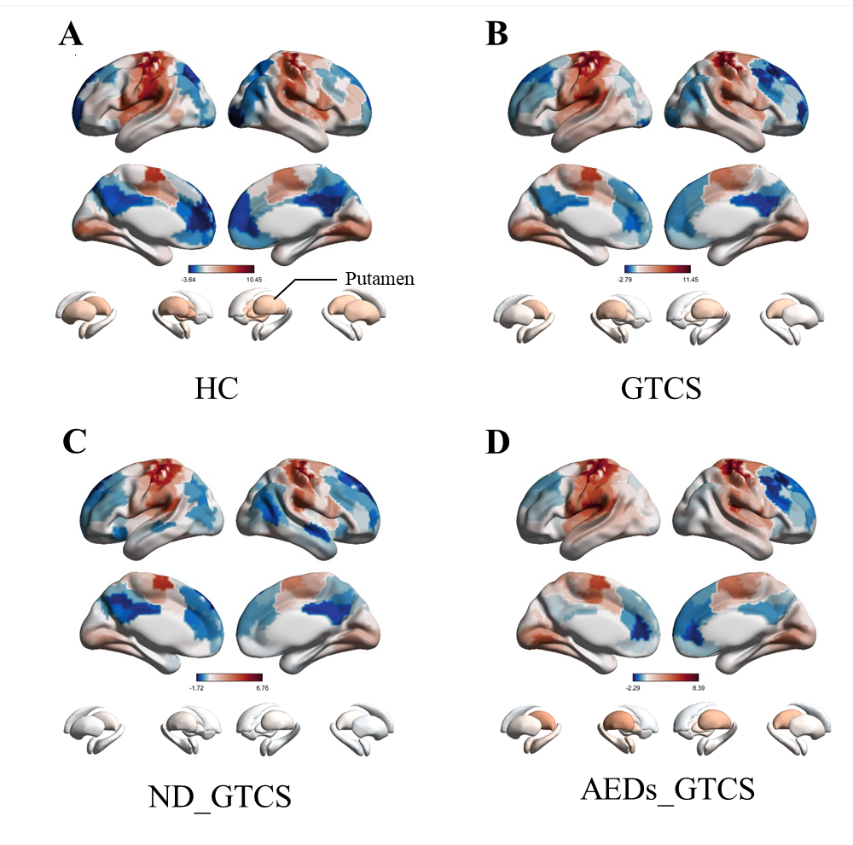


**Fig. S2** Motor task activation maps in each group using one sample t-test (unthresholded).


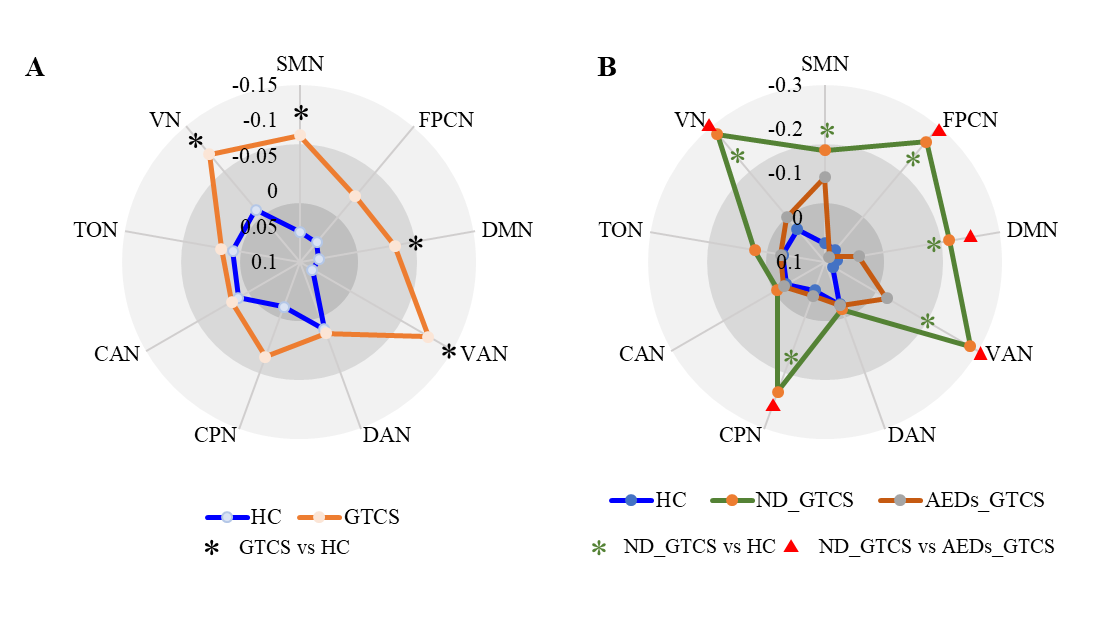


**Fig. S3** Comparisons of association between motor activation and head motion. (A) Comparisons between GTCS and HC. (B) Comparisons between AEDs_GTCS, ND_GTCS, and HC.


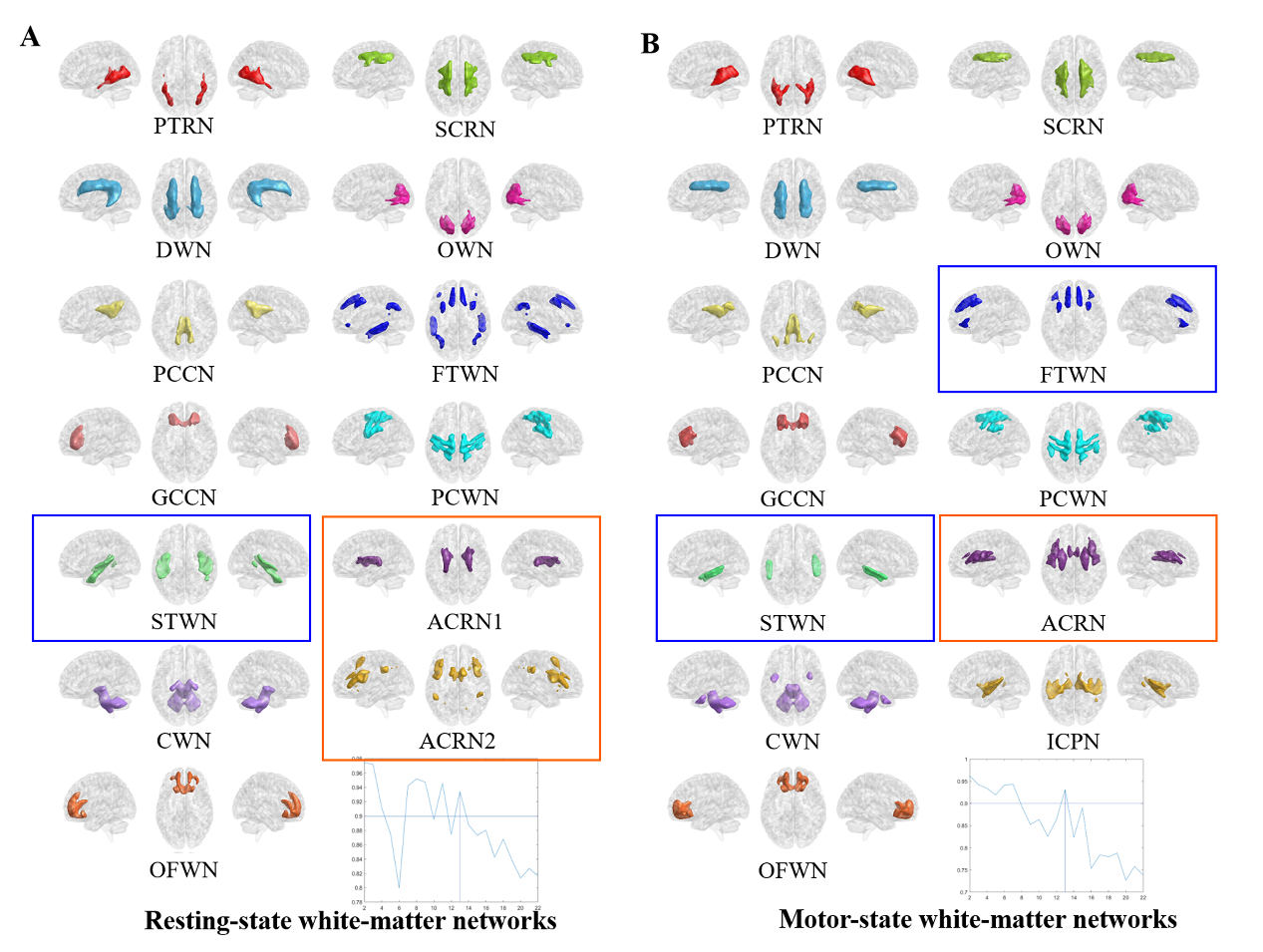


**Fig. S4** White-matter networks clustered using resting and motor-task state fMRI separately. Thirteen white-matter networks were identified in the resting state (A) and motor-task state (B). Blue and yellow boxes represent white-matter networks different across states.


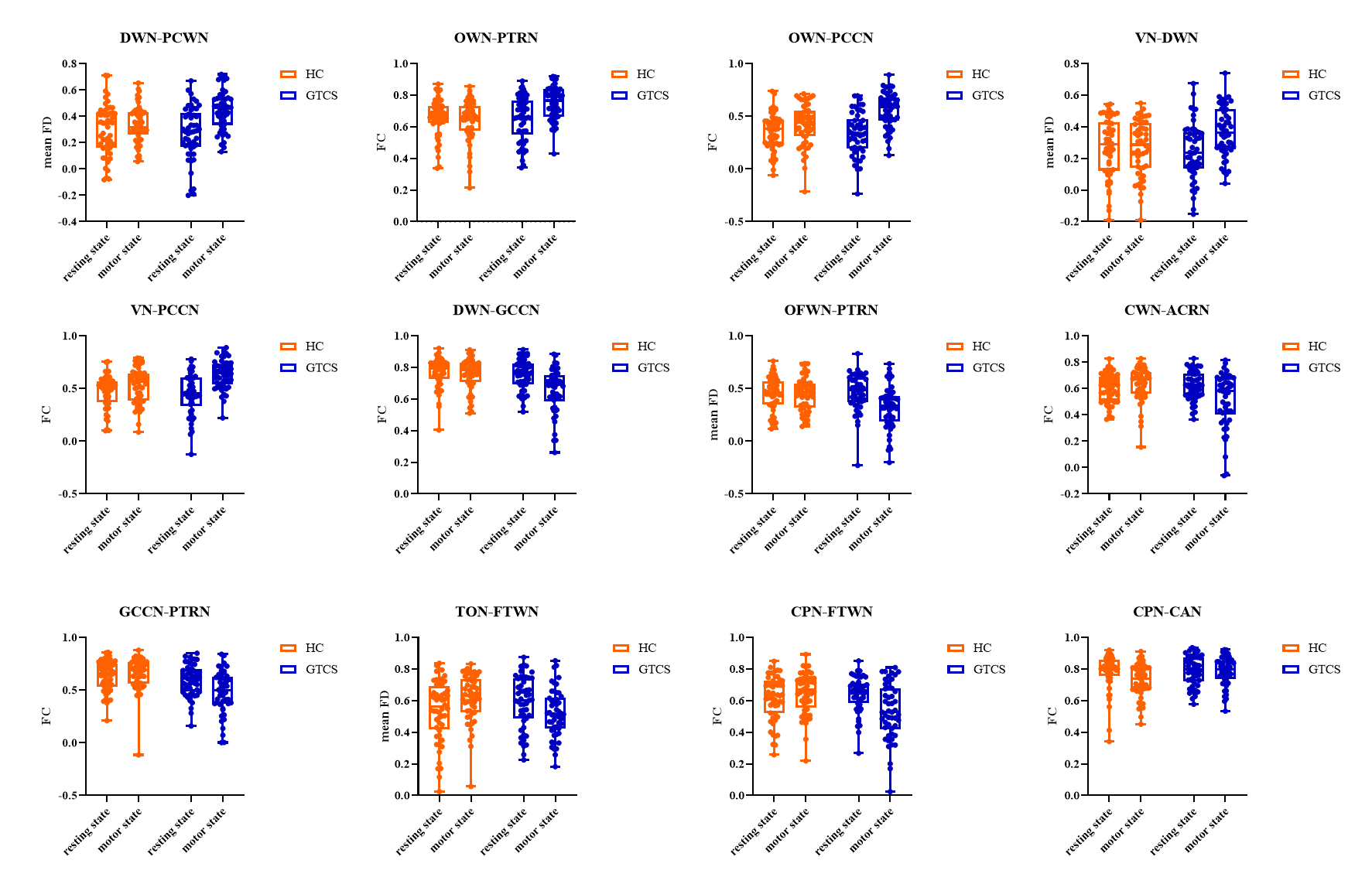


**Fig. S5** Connections among ND_GTCS, AEDs_GTCS, and HC between states (each dot represent a subject).


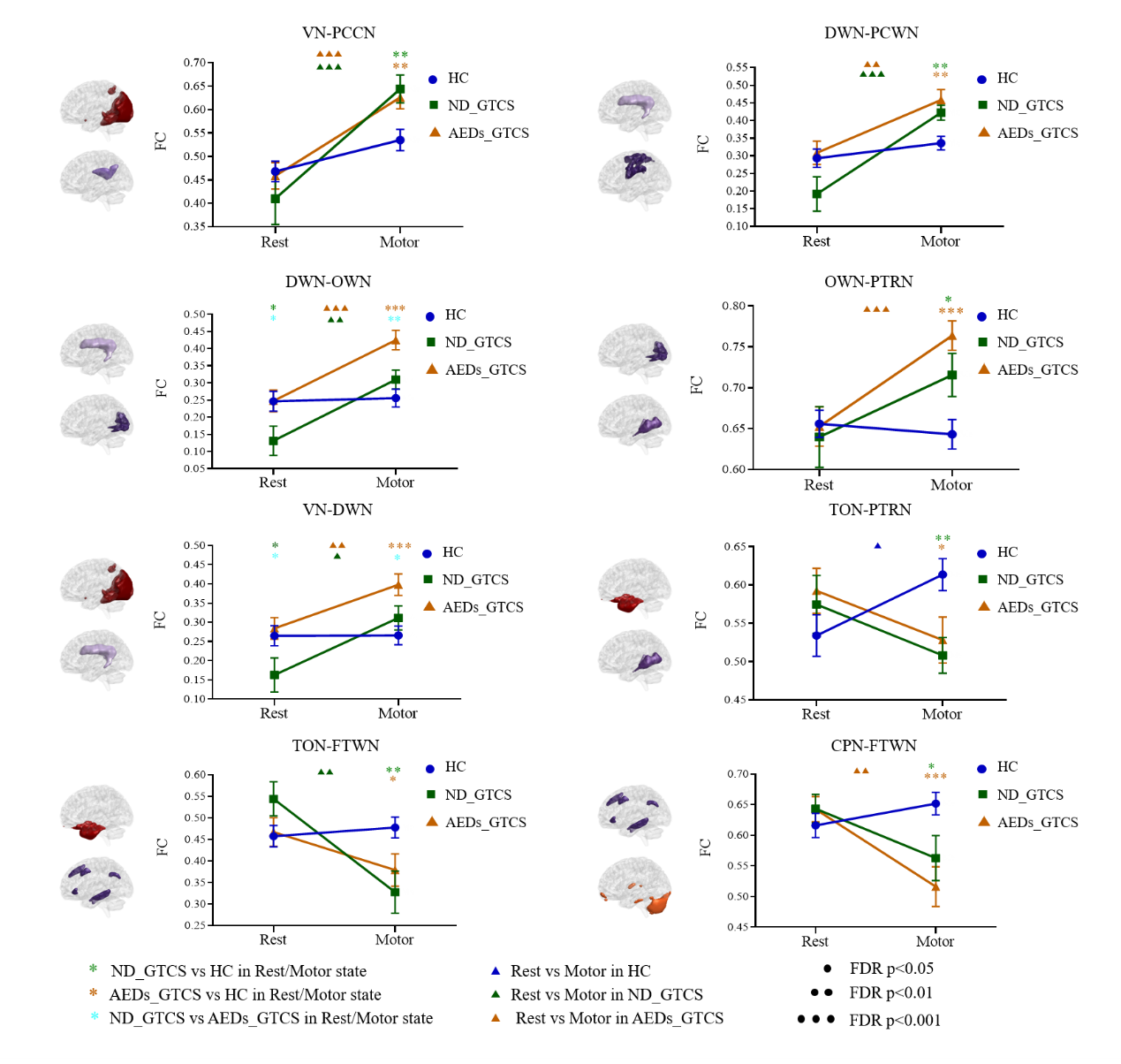


**Fig. S6** Connections comparisons among ND_GTCS, AEDs_GTCS, and HC between states.


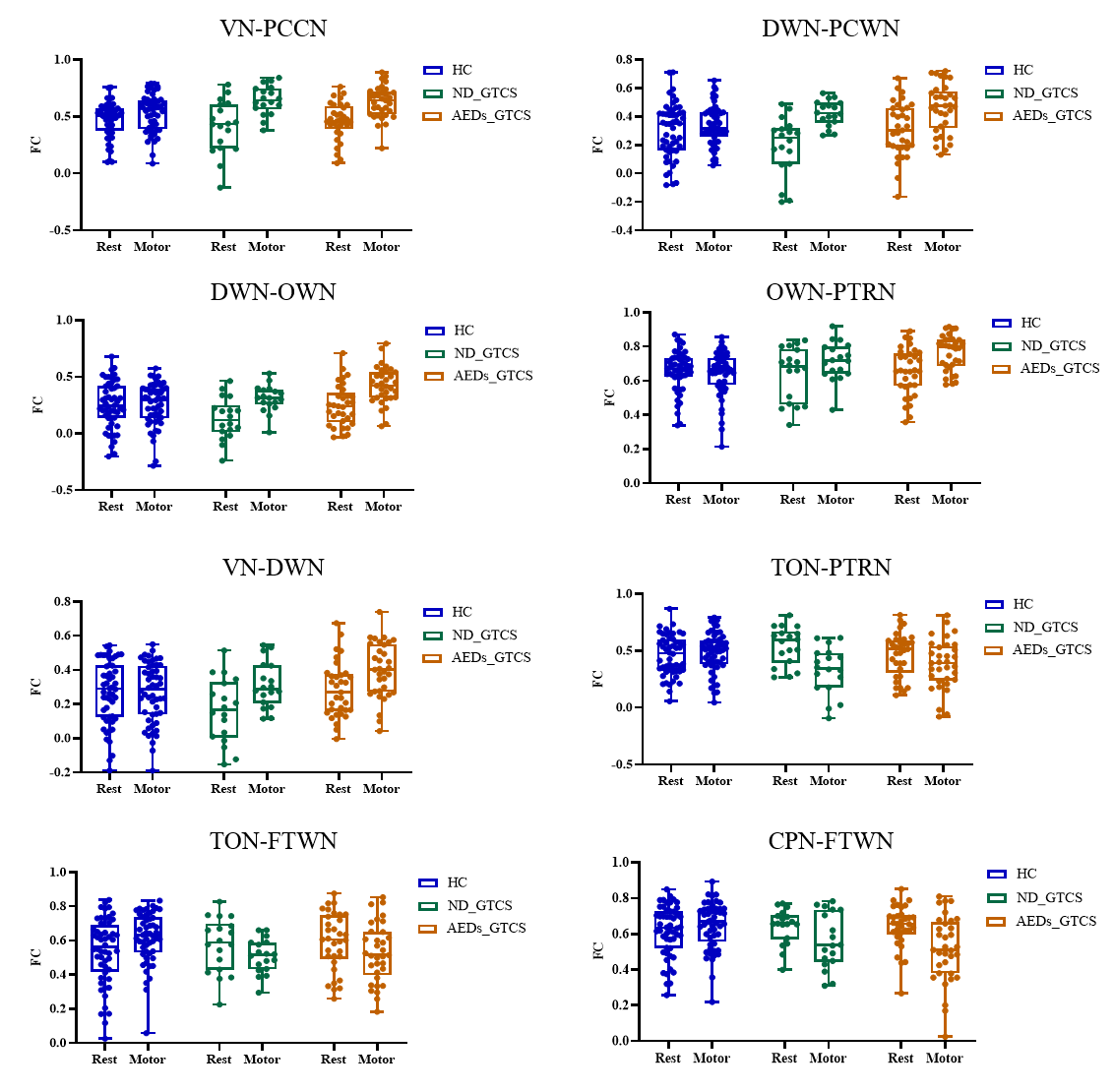


**Fig. S7** Connections among ND_GTCS, AEDs_GTCS, and HC between states (each dot represent a subject).

Table S1. Difference of motion task activation

| Brain region | MNI coordinates  (X Y Z) | BA | t-value | p-value |
| --- | --- | --- | --- | --- |
| **GTCS vs. HC** |  |  |  |  |
| Putamen L | -25 -2 10 | 48 | -3.21 | 9e-4 |
| Putamen R | 24 6 9 | 48 | -3.07 | 1e-3 |
| Postcentral R | 26 -38 64 | 2 | 4.28 | 2e-5 |
| Postcentral L | -29 -41 55 | 3 | 3.77 | 1e-4 |
| **ND_GTCS vs. HC** |  |  |  |  |
| Putamen L | -3 -45 -69 | 48 | -3.01 | 1e-3 |
| Putamen R | 32 -2 5 | 48 | -2.90 | 2e-3 |
| Postcentral L | -32 -43 56 | 3 | 3.12 | 1e-3 |
| **AEDs_GTCS vs. HC** |  |  |  |  |
| Postcentral R | 25 -31 63 | 2 | 4.03 | 6e-5 |
| Supplementary motor area | 4 -15 64 | 6 | 3.24 | 8e-4 |
| Postcentral L | -29 -39 57 | 3 | 3.15 | 1e-3 |

Table S2. White-matter functional networks.

| Number | White-matter network | Abbreviation | Layer |
| --- | --- | --- | --- |
| 1 | Posterior thalamic radiation network | PTRN | Middle |
| 2 | Deep white-matter network | DWN | Deep |
| 3 | Posterior corpus callosum network | PCCN | Middle |
| 4 | Genu of corpus callosum network | GCCN | Middle |
| 5 | Superior temporal white-matter network | STWN | Superficial |
| 6 | Cerebellar white-matter network | CWN | Middle |
| 7 | Orbitofrontal white-matter network | OFWN | Superficial |
| 8 | Superior corona radiate network | SCRN | Middle |
| 9 | Occipital white-matter network | OWN | Superficial |
| 10 | Frontotemporal white-matter network | FTWN | Superficial |
| 11 | Precentral/Postcentral white-matter network | PCWN | Superficial |
| 12 | Anterior corona radiate network | ACRN | Middle |

Table S3 Decreased AS-FC in ND_GTCS, AEDs_GTCS relative to HC.

| Resting-state network | Motor-task state network | t-value | p-value |
| --- | --- | --- | --- |
| **ND_GTCS vs. HC** |  |  |  |
| Cerebellum anterior network | Frontoparietal control network | -3.04 | 2e-3 |

Table S4 Decreased AS-FC and AS-HFC in patients relative to the HC.

| Resting-state network | Motor-task state network | t-value | p-value |
| --- | --- | --- | --- |
| **AS-FC** |  |  |  |
| Cerebellum anterior network | Frontoparietal control network | -3.41 | 5e-4 |
| **AS-HFC** |  |  |  |
| Ventral attention network | Cerebellum anterior network | -3.59 | 1e-4 |
|  | Cerebellar white-matter network | -3.78 | 1e-4 |
| Cerebellum posterior network | Orbitofrontal white-matter network | -3.66 | 2e-4 |
| Cerebellum anterior network | Orbitofrontal white-matter network | -3.60 | 3e-4 |
| Visual network | Orbitofrontal white-matter network | -3.90 | 9e-5 |
| Genu of corpus callosum network | Cerebellum posterior network | -3.74 | 2e-4 |
| Frontotemporal white-matter network | Frontotemporal white-matter network | -3.53 | 3e-4 |
|  | Cerebellum posterior network | -4.59 | 7e-6 |
| Anterior corona radiate network | Cerebellum posterior network | -3.55 | 3e-4 |

Table S5 Decreased AS-HFC in ND_GTCS, AEDs_GTCS relative to HC.

| Resting-state network | Motor-task state network | t-value | p-value |
| --- | --- | --- | --- |
| **ND_GTCS vs. HC** |  |  |  |
| Ventral attention network | Cerebellum anterior network | -4.49 | 5e-5 |
| Ventral attention network | Cerebellar white-matter network | -4.26 | 3e-5 |
| Cerebellum posterior network | Orbitofrontal white-matter network | -3.52 | 4e-4 |
| Cerebellum anterior network | Orbitofrontal white-matter network | -3.72 | 2e-4 |
| Orbitofrontal white-matter network | Orbitofrontal white-matter network | -3.30 | 8e-4 |
| Frontotemporal white-matter network | Cerebellum posterior network | -3.24 | 9e-4 |
| **AEDs_GTCS vs. HC** |  |  |  |
| Ventral attention network | Cerebellum anterior network | -4.89 | 2e-6 |
| Cerebellum posterior network | Orbitofrontal white-matter network | -3.48 | 4e-4 |
| Cerebellum anterior network | Orbitofrontal white-matter network | -3.66 | 2e-4 |
| Visual network | Orbitofrontal white-matter network | -3.08 | 1e-3 |
| Orbitofrontal white-matter network | Orbitofrontal white-matter network | -3.85 | 1e-4 |
| Frontotemporal white-matter network | Cerebellum posterior network | -3.51 | 3e-4 |
| Anterior corona radiate network | Cerebellum posterior network | -3.50 | 3e-4 |
